# Identification of differences in human and great ape phytanic acid metabolism that could influence gene expression profiles and physiological functions

**DOI:** 10.1186/1472-6793-10-19

**Published:** 2010-10-08

**Authors:** Paul A Watkins, Ann B Moser, Cicely B Toomer, Steven J Steinberg, Hugo W Moser, Mazen W Karaman, Krishna Ramaswamy, Kimberly D Siegmund, D Rick Lee, John J Ely, Oliver A Ryder, Joseph G Hacia

**Affiliations:** 1 Hugo W. Moser Research Institute at Kennedy Krieger, and Department of Neurology, Johns Hopkins University School of Medicine, Baltimore, MD, 21205, USA; 2 Department of Biochemistry and Molecular Biology, University of Southern California, Los Angeles, CA, 90089, USA; 3 Department of Preventive Medicine, University of Southern California, Los Angeles, CA 90089, USA; 4 Alamogordo Primate Facility, New Mexico, NM 88330, USA; 5 Institute for Conservation and Research, Zoological Society of San Diego, Escondido, CA 92027, USA

## Abstract

**Background:**

It has been proposed that anatomical differences in human and great ape guts arose in response to species-specific diets and energy demands. To investigate functional genomic consequences of these differences, we compared their physiological levels of phytanic acid, a branched chain fatty acid that can be derived from the microbial degradation of chlorophyll in ruminant guts. Humans who accumulate large stores of phytanic acid commonly develop cerebellar ataxia, peripheral polyneuropathy, and retinitis pigmentosa in addition to other medical conditions. Furthermore, phytanic acid is an activator of the PPAR-alpha transcription factor that influences the expression of genes relevant to lipid metabolism.

**Results:**

Despite their trace dietary phytanic acid intake, all great ape species had elevated red blood cell (RBC) phytanic acid levels relative to humans on diverse diets. Unlike humans, chimpanzees showed sexual dimorphism in RBC phytanic acid levels, which were higher in males relative to females. Cultured skin fibroblasts from all species had a robust capacity to degrade phytanic acid. We provide indirect evidence that great apes, in contrast to humans, derive significant amounts of phytanic acid from the hindgut fermentation of plant materials. This would represent a novel reduction of metabolic activity in humans relative to the great apes.

**Conclusion:**

We identified differences in the physiological levels of phytanic acid in humans and great apes and propose this is causally related to their gut anatomies and microbiomes. Phytanic acid levels could contribute to cross-species and sex-specific differences in human and great ape transcriptomes, especially those related to lipid metabolism. Based on the medical conditions caused by phytanic acid accumulation, we suggest that differences in phytanic acid metabolism could influence the functions of human and great ape nervous, cardiovascular, and skeletal systems.

## Background

Humans and great apes (bonobos, chimpanzees, gorillas, and orangutans) share a common gut anatomy, consisting of a simple stomach, small intestine, small cecum terminating in an appendix, and a hindgut consisting of the large intestine, rectum, and anal canal [1]. Nevertheless, significant differences have been reported in their gut proportions [2-4]. While the large intestine represents the majority of the great ape gut volume, the majority of the modern human gut volume consists of the small intestine [2-4]. Initial surveys have also indicated modern humans have a smaller total gut volume to body mass ratio relative to the great apes [5-7]. However, this could be influenced by primate gut plasticity related to diet and genetic diversity [2,8].

It has been proposed that gut proportions changed at some point within the human lineage in response to higher quality foods which can be digested in the small intestine [2]. The diets of hominids and/or early human populations improved, in part, due to cooking [9] and the increased abundance of animal products obtained through scavenging, hunting, fishing, and dairy consumption [10-19]. In contrast, great ape species in the wild derive a significant amount of their total daily metabolic energy needs through the fermentation of lower quality plant materials in their hindguts [20-25]. Although hindgut fermentation also occurs in humans [26-28], there is evidence that wild great apes derive greater amount of total daily metabolic energy from this process than do humans on Western diets [20-22]. However, seasonal changes in great ape diets and the limited dietary diversity of the humans studied will influence the interpretation of these data sets.

To explore the systemic consequences of hindgut fermentation activities in humans and great apes, we evaluated their physiological levels and cellular metabolic activities of phytanic acid, a branched chain fatty acid that can bind to and/or activate PPAR-alpha [29-34] and RXR [35-38] transcription factors. Humans do not synthesize phytanic acid, but rather acquire it from the diet [39,40]. In ruminants, the gut fermentation of plant materials liberates phytol, a constituent of chlorophyll, which is then converted to phytanic acid and stored in fats [39,40]. While humans can covert free phytol into phytanic acid, they do not accumulate significant amounts of phytanic acid as a result of consuming plant materials [41,42]. However, they can obtain phytanic acid from ruminant fats, fish, and dairy products [41]. Humans with impaired phytanic acid catabolism can overaccumulate phytanic acid, which results in peripheral polyneuropathy, cerebellar ataxia, retinitis pigmentosa, anosmia, and hearing loss [43-45]. This can also result in cardiac arrhythmias, shortened metacarpals or metatarsals, and ichthyosis [43-45].

Despite the demonstrated importance of maintaining appropriate levels of phytanic acid in humans and mice [46-48], phytanic acid metabolism has not been studied in the great apes. Based on the biochemical evaluation of red blood cells and cultured primary skin fibroblasts, we uncovered candidate human-specific differences in the ability to obtain phytanic acid from dietary sources relative to the great apes. We also provide indirect evidence that these changes could affect physiological functions relevant to the evolution and health of these species.

## Results

### Red blood cell (RBC) phytanic acid levels

**Figure 1:**
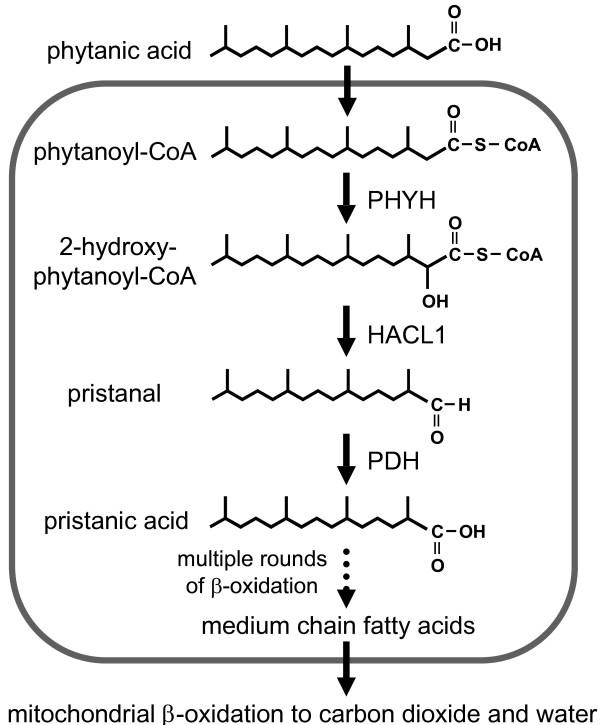
**Phytanic acid catabolism in mammals**. Phytanic acid in ruminant fats is derived from phytol produced during the bacterial degradation of chlorophyll in their rumen (first stomach). After conversion to its CoA thioester, phytanic acid undergoes α-oxidation, yielding pristanic acid. This fatty acid then undergoes three subsequent rounds of β-oxidation in the peroxisome. The resulting medium chain fatty acid exits the peroxisome and translocates to the mitochondrion where the remaining carbon chain is degraded by β-oxidation. Abbreviations for the enzymes listed include: HACL1 (*aka *HPCL2) = 2-hydroxyphytanoyl-CoA lyase; PHYH = phytanoyl-CoA α-hydroxylase; PDH = pristanal dehydrogenase, whose gene is not yet known. We note that phytanic acid can also be degraded by β-oxidation; however, this activity of this pathway is relatively minor [40,44].

**Figure 2:**
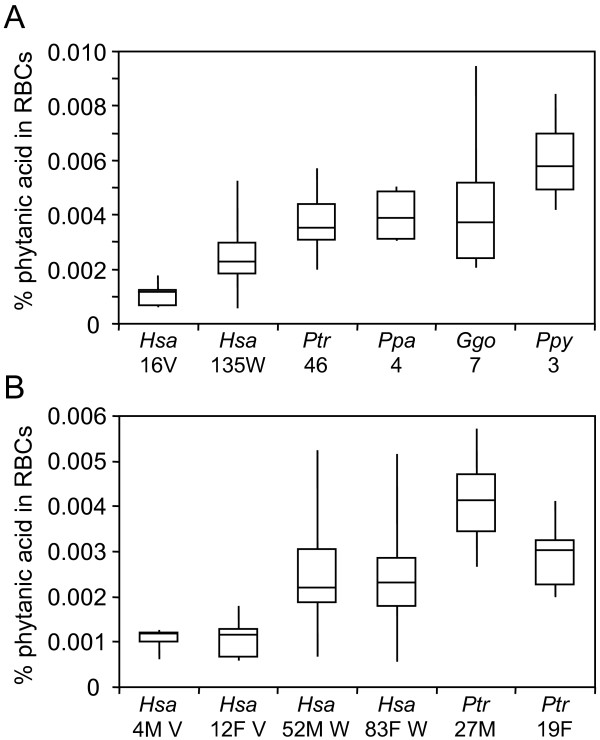
**Phytanic acid levels in human and great ape red blood cells**. Box plots representing the percentage of phytanic acid relative to total fatty acids from red blood cells are provided. Median, quartile 1, quartile 3, minimum, and maximum values are provided. In Panels A and B, the species (*Hsa*: human; *Ptr*: chimpanzee; *Ppa*: bonobo; *Ggo*: gorilla; *Ppy*: orangutan), human diet (V: vegan, W: western), and number of individuals successfully analyzed is provided on the X-axes. Blood donor sex (M: male, F: female) is provided in Panel B.

RBCs are commonly used to evaluate the physiological abundance of phytanic acid (Fig. 1) in clinical settings [49]. First, we measured RBC phytanic acid levels in a cohort of humans with Western diets (N = 135), chimpanzees (N = 46), bonobos (N = 4), lowland gorillas (N = 7), and Sumatran orangutans (N = 3) (Fig. 2A and Additional File 1). We found significant differences in RBC phytanic acid levels in humans with Western diets relative to the great apes (*P *< 2×10^-13^). Chimpanzee (1.5-fold, *P *< 3 × 10^-10^), bonobo (1.7-fold, *P *< 6 × 10^-3^), gorilla (1.7-fold, *P *< 7 × 10^-4^), and orangutan (2.6-fold, *P *< 4 × 10^-5^) RBC phytanic acid levels were all elevated relative to those of this human cohort. This is remarkable given the higher phytanic acid content of human Western diets, reportedly 50-100 mg per day [43], relative to the minimal (< 2 mg) daily amounts in the diets of our great ape cohort, even taking nutritional biscuits into consideration as described in the Materials and Methods section.

To begin to address these dietary differences, we compared RBC phytanic acid levels in great apes and humans on phytanic acid-deficient diets (Fig. 2A). The latter are based on a cohort of 16 humans on vegan diets for over one year (Additional File 1). The absolute amount of phytanic acid intake in this population is unknown; however, it should be minimal (< 10 mg), based on typical components of vegan diets [41,50]. Phytanic acid RBC levels were lower in vegans relative to individuals on Western diets (2.3-fold, *P *< 1 × 10^-13^). We also found robust differences in vegan RBC phytanic acid levels relative to the great apes (*P *< 3 × 10^-25^). RBC phytanic acid levels in chimpanzees (3.5-fold, *P *< 6 × 10^-20^), bonobos (3.9-fold, *P *< 6 × 10^-10^), gorillas (3.8-fold, *P *< 2 × 10^-12^), and orangutans (5.9-fold, *P *< 5 × 10^-12^) were all dramatically higher relative to vegans.

Given the composition of our cohort, we had adequate statistical power to screen for possible sexual dimorphism in human (Western diet) and chimpanzee RBC phytanic acid levels. Although no sex-specific differences were found in humans on Western diets, male chimpanzees showed 1.4-fold higher (*P *< 2 × 10^-6^) RBC phytanic acid levels relative to females (Fig. 2B). This occurs despite the fact that our cohort of male and female chimpanzees had equivalent dietary exposure to phytanic acid and its precursor, phytol. Although our power is limited due to the numbers of vegan males in our cohort, there was also no evidence of sexual dimorphism in vegan RBC phytanic acid levels (Fig. 2B).

### Phytanic acid catabolism in cell culture

**Figure 3:**
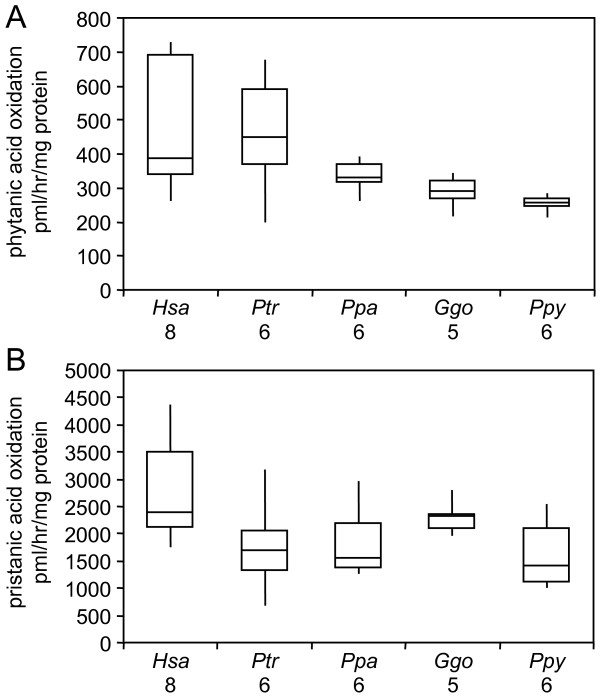
**Phytanic acid catabolism in human and great ape cultured fibroblasts**. Box plots representing the relative rates of (A) phytanic acid and (B) pristanic acid oxidation in cultured fibroblasts are provided. Median, quartile 1, quartile 3, minimum, and maximum values are provided. The species and number of samples successfully analyzed is provided on the X-axis of both panels.

To evaluate cellular rates of phytanic acid metabolism, we analyzed cultured human and great ape dermal fibroblasts (Additional File 2), which are routinely used for the clinical diagnoses of phytanic acid and other peroxisomal disorders [43]. All fibroblast cultures showed robust catabolism of phytanic acid and its metabolite pristanic acid. Nevertheless, the oxidation rates of phytanic acid (*P *= 0.010) and pristanic acid (*P *= 0.048) differed in human relative to great ape cells (Fig. 3A-B). Human cells showed elevated phytanic acid oxidation rates relative to gorilla (1.6-fold, *P *= 0.017) and orangutan (1.8-fold, *P *= 0.002) cells (Fig. 3A). Humans cells also showed elevated pristanic acid oxidation rates compared to chimpanzee (1.7-fold, *P *= 0.018), bonobo (1.5-fold, *P *= 0.05), and orangutan (1.8-fold, *P *= 0.011) cells (Fig. 3B).

### Comparative transcriptome analysis of peroxisomal α- and β-oxidation pathways

**Figure 4:**
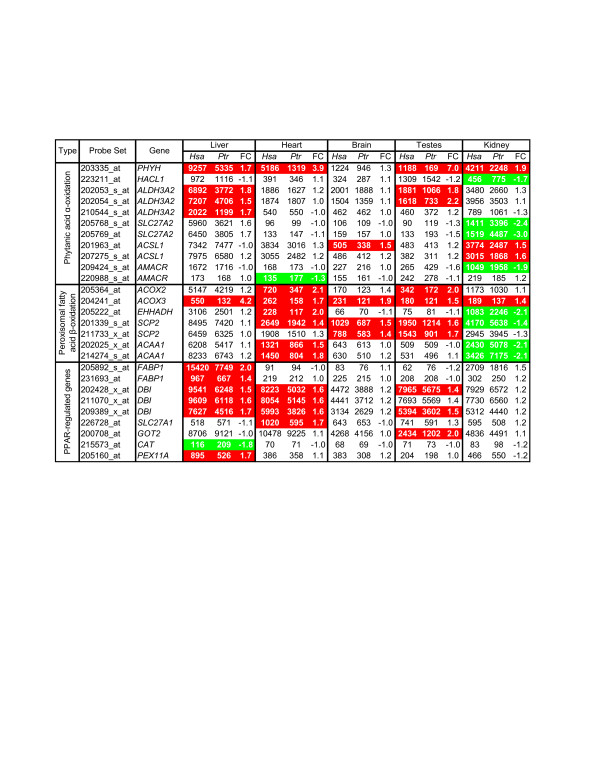
**Differential expression of genes related to peroxisomal lipid metabolism**. We reanalyzed of Affymetrix GeneChip U133v2.0 expression profiles of human and chimpanzee tissues [51] using the masking strategy stated in the text. We used standard F-tests (FDR-adjusted using the Benjamini and Hochberg approach) to test for differences in the distributions by species for the 5 tissues. The fold change (FC) of human (*Hsa*) versus chimpanzee (*Ptr*) geometric mean gene expression scores are provided. Differentially expressed genes (≥1.2 FC in either direction with a Student's t-test, two-tailed P-value ≤0.05 after Bonferroni correction) for a given tissue are highlighted in red (higher in human) or green (higher in chimpanzee). Probe sets with (i) F-tests yielding a ≤5% FDR and (ii) differential expression in at least one tissue are shown. All data are provided in Additional File 3.

To evaluate potential *in vivo *consequences of cross-species differences in lipid metabolism, we reanalyzed oligonucleotide microarray gene expression data from 5 male chimpanzee and 6 male human livers, brains, kidneys, heart, and testes [51]. We only considered data from probes predicted to be perfectly matched to both genomes and calculated gene expression scores only for probe sets containing at least four probes after masking [52]. We focused on genes involved in peroxisomal phytanic acid α-oxidation, β-oxidation, and PPAR-responsive genes relevant to phytanic acid metabolism (Fig. 4, Additional File 3). Our criteria for differential gene expression are provided in the legend of Figure 4.

We found that cross-species differential expression of phytanic acid α-oxidation and peroxisomal β-oxidation genes in the liver, brain, and testes always reflected higher transcript levels in humans (Fig. 4). Genes in these categories also showed cross-species differential expression in heart and kidney; however, some transcripts were more abundant in humans (7 for heart and 4 for kidneys) while others were more abundant in chimpanzees (1 for heart and 8 for kidneys). Strikingly, *ACOX3 *transcript levels were higher in humans relative to chimpanzees for all five tissues examined. In 4 tissues (liver, heart, testes, and kidney), *PHYH *transcript levels were also higher in human relative to chimpanzee. To further explore this latter observation, we measured *PHYH *levels in human, chimpanzee, bonobo, and gorilla fibroblast cultures (6 different donors per species) via quantitative PCR (qPCR) using primers designed against a conserved region of this transcript. *PHYH *levels were at least 2-fold more abundant in human relative to chimpanzee, bonobo, and gorilla fibroblasts (*P *< 3 × 10^-4 ^for each individual comparison, ANOVA *P *< 2 × 10^-5 ^for human versus African great apes).

Lastly, we examined a group of five genes (*FABP1*, *DBI*, *SLC27A1*, *GOT2*, and *CAT*) previously used to evaluate PPAR-activity in mice with phytanic acid disorders [53] and *PEX11A*, a direct transcriptional target of PPAR-alpha [54]. All differentially expressed transcripts in heart (*DBI *and *SLC27A1*) and testes (*DBI *and *GOT2*) were more abundant in humans. *FABP1*, *DBI*, and *PEX11A *transcript levels were higher in human liver; however, *CAT *transcripts were more abundant in chimpanzee liver. None of these six genes showed cross-species differential expression in brain or kidney.

## Discussion

Microbial fermentation in human [26-28] and great ape [20-25] hindguts are responsible for the break down of complex carbohydrates not processed and absorbed in the small intestine. The major end products of hindgut fermentation are short chain fatty acids, which provide energy-yielding substrates for colonic mucosa that regulate its growth and blood flow and promotes sodium and water absorption [26,27]. It has been estimated that wild chimpanzees derive 21-33% and wild orangutans derive 7-57% of their total daily metabolic energy from fiber fermentation [22]. This could be as high as 30-60% for wild Western gorillas [21]. In contrast, humans on Western diets are estimated to obtain no more than 10% of their daily energy needs through hindgut fermentation; however, this is likely higher in populations with lower quality diets and instances of small intestinal malabsorption [28].

Although we contrast human and the great ape dietary strategies, it is important to consider diversity within the great apes. For example, gorillas and orangutans can and often do subsist on lower quality foods (e.g. mature foliage and unripe fruits) relative to chimpanzees and bonobos, which can consume ample amounts of succulent ripe fruits [2]. Furthermore, chimpanzees hunt colobus monkeys and other small mammals; however, this comprises a minor component of their diets and varies among individuals and communities [55-59]. Thus, we propose that it is likely that the human, chimpanzee, bonobo, gorilla and orangutan lineages have all acquired molecular adaptations relevant to their diets.

In the studies presented here, we began to evaluate the broader systemic consequences of hindgut fermentation and related lipid metabolic activity in humans and great apes. In principle, RBC phytanic acid can be an effective biomarker of hindgut fermentation of plant materials as long as phytanic acid or its precursor phytol is not appreciably present in the human or great ape diets. The phytanic acid content of Western diets (50-100 mg daily intake) [43] is estimated to be at least 10 × greater than its free phytol content [41,60]. Since phytanic acid is primarily found in ruminant fats (organic beef fat has >325 mg/100 g), dairy products (45% fat cream cheese has >125 mg/100 g), and fish (canned salmon has >250 mg/100 g) [41], human vegans are appropriate for our studies. In this regard, a survey of fresh vegetables and fruits all showed minimal phytol levels (≤2 mg phytol/100 g) [41]. We also note a prior study demonstrating that an individual who ingested 3.5-kg of boiled spinach leaves within a 60 hour period as a supplement to an uncontrolled mixed diet later showed serum phytanic acid levels within normal limits [42]. Our captive great ape cohort should be exposed to similar low levels of dietary phytanic acid (< 2 mg daily intake) and phytol [41].

We discovered elevated RBC phytanic acid levels in all great apes relative to humans on both high- (Western) and low-phytanic acid (vegan) diets (Fig. 2). In principle, these could be influenced by differences in phytanic acid retention and/or biosynthesis. Relevant to the retention hypothesis, both humans and great apes showed robust cellular rates of phytanic and pristanic acid oxidation (Fig. 3). In fact, there were no differences in phytanic acid oxidation rates in human relative to chimpanzee or bonobo fibroblasts (Fig. 3A). Although it is possible that the lower phytanic acid oxidation rates in gorilla and orangutan relative to human fibroblasts (Fig. 3A) relate to the dietary strategies of these species [2], these observations need to be validated in larger-scale studies prior to commentary. We also cannot exclude the possibility that the cultured fibroblasts do not fully reflect *in vivo *activities; however, they are invaluable for the clinical diagnosis of phytanic acid oxidation disorders [61]. Likewise, we cannot exclude the possibility that great apes, unlike humans, show robust biological retention of trace dietary phytanic acid. However, laboratory mice do not accumulate significant physiological levels of phytanic acid when fed standard diets not supplemented with additional phytol or phytanic acid [53]. In addition, rats on standard diets can rapidly catabolize artificially elevated stores of phytanic acid in their tissues [62].

Our preferred hypothesis is that all the great apes, unlike humans [45,63], are capable of accumulating significant amounts of phytanic acid from the hindgut fermentation of plant materials. We propose that the great apes have maintained the ancestral condition and that humans diverged since their most recent common ancestor with chimpanzees and bonobos and now have a derived metabolism. We believe that changes in gut anatomy provide a likely basis for the proposed reduction of hindgut fermentation activity in the human lineage. In this regard, it has been proposed that improved dietary quality contributed to hindgut reduction in modern humans relative to great apes (i.e. the hindgut comprises ~20% of the total human gut volume, but >50% of the total great ape gut volume) [2,3] and the smaller total gut volume to body mass ratio in modern humans relative to great apes [6,7,64,65]. However, we also recognize that the human and great ape gut microbiomes, whose composition are influenced by the diets of these species and other environmental interactions [66,67], could also contribute to the proposed differences in hindgut fermentation activity. Thus, in principle, the improved dietary quality in the human lineage could have influenced the major factors (gut anatomy and microbial activities) that we propose are most likely to be causally responsible for the observed differences in human and great ape RBC phytanic acid levels.

Given that sexual dimorphism in human lipid metabolism is well-documented [68,69], we screened for sex-specific differences in RBC phytanic acid levels. Here, we found no evidence of sexual dimorphism in RBC levels in humans on Western or vegan diets. However, we observed 1.4-fold elevated RBC phytanic acid levels in male relative to female chimpanzees (Fig. 2B). The evolutionary implications and physiological origin of this sexual dimorphism are unclear. Phytanic acid profiling in additional males and females from other non-human primates could shed light on the extent of this phenomenon. As discussed above, phytanic acid levels are affected by multiple factors, including gut anatomies, microbiomes, and gene expression, which provide candidate mechanisms for the observed sexual dimorphism in chimpanzees.

We note that sexual dimorphism in phytanic acid metabolism has been documented in rodents. C57BL/6J laboratory mice show sexually dimorphic phytanic acid accumulation in response to phytol-enriched diets; however, unlike chimpanzees, females showed higher phytanic acid levels than males [53,70]. There is no evidence as to whether this is a metabolic adaptation to diet or a non-specific result of inbreeding. It is thought this sexual dimorphism is influenced by the higher liver expression of sterol carrier protein-x (SCP-x) in males relative to females [53]. SCP-x is required for branched chain fatty acid catabolism through its keto-acyl CoA thiolase activity [53,70]. Sexual dimorphism in liver SCP-x expression has also been observed in FVB mice [53], BALB/c mice [71], and laboratory rats [72]. This suggests that sexual dimorphism in phytanic acid metabolism could be present in additional rodent species. To date, SCP-x expression has not been measured in human and chimpanzee livers, even in a recent study involving both sexes from each species [73].

We also investigated possible relationships between human and chimpanzee RBC lipid profiles and their transcriptomes. While there is no known physiological role for phytanic acid, it can bind to and/or activate PPAR-alpha [29-34], RXR [35-38], and likely other transcription factors [29]. Experiments involving laboratory mice have concluded that phytanic acid is a physiological ligand for PPAR-alpha [29-31]. While the estimated phytanic acid concentration in normal human serum (6 μM total [42], 2 μM free [35]) was at the threshold needed for RXR stimulation of one cell culture study, the authors concluded that phytol metabolites, such as phytanic acid, are compelling candidate physiological effectors of RXR [35].

Here, we found that multiple genes reported to be influenced by PPAR-alpha activity, including those involved in peroxisomal α- and β-oxidation of fatty acids, show cross-species expression differences in the chimpanzee and human tissues surveyed (Fig. 4). Such genes were primarily more highly expressed in humans relative to chimpanzee for all tissues except kidney, where higher expression in chimpanzees was frequently found. Exploratory work, not involving non-human primates, highlighted the lower fatty acid α-oxidation capacity in the kidneys, but not livers, of carnivorous relative to herbivorous animals [74]. Also of interest, others have provided gene expression-based evidence that PPAR-signaling is higher in human relative to chimpanzee liver [75]. The elevated *FABP1 *transcript levels in human liver suggests an increased capacity for humans to transport phytanic acid to peroxisomes for degradation [53]. Nevertheless, we recognize phytanic acid is just one of several activators of PPAR transcription factors and that the dietary information and RBC lipid profiles of the humans and chimpanzees in the gene expression profiling studies are unknown.

We have previously noted that the toxicity associated with abnormal phytanic acid accumulation in humans could provide selective pressure to maintain or enhance its degradation [76]. For example, Refsum disease is an autosomal recessive disorder in humans that can result from mutations in the *PHYH *gene and subsequent accumulation of phytanic acid in tissues [46,47]. This typically affects the peripheral nerves (i.e. polyperipheral neuropathy), movement (i.e. cerebellar ataxia), and senses (e.g. retinitis pigmentosa, anosmia, and hearing loss) [43-45]. These individuals can also show cardiac arrhythmias, skeletal malformations (e.g. shortened metacarpals or metatarsals), and skin changes (i.e. ichthyosis) [43-45]. Given that cardiac arrhythmias are suspected to be responsible for the sudden deaths of Refsum disease patients [43], the commonly observed sudden cardiac death, presumed secondary to fatal arrhythmias, in captive male chimpanzees [77] could be influenced by their physiological levels of phytanic acid. However, the RBC phytanic acid levels in our cohort of male chimpanzees are far below those of Refsum disease patients, which was approximately 100 times higher than normal in one case we evaluated. Nevertheless, we raise the possibility that phytanic acid metabolism could influence physiological differences and medical conditions amongst human and non-human primates.

## Conclusions

The candidate differences in human and great ape phytanic acid metabolism are consistent with prior reports that the regulation of genes involved in lipid metabolism likely evolved under directional selection in humans [75] and that the promoter regions of nutrition-related genes are under positive selection in humans [78]. It is also relevant that the effects human diets have on mouse liver transcriptomes can be used as a model for expression differences between humans and chimpanzees [79]. We note that even if transcriptome data of humans and chimpanzees on matching diets were obtained, differences in how each species digests their food and extract regulators of transcription would have a major impact on the observed cross-species and sex-specific differences.

Our study also relates to hypotheses that historic diets and/or changes in lipid metabolism influenced the evolution of numerous human traits [2,12,18,55,64,65,80-85]. To further explore such hypotheses, it will be necessary to determine if the RBC phytanic acid profiles in our cohort extend to other cell types. The comparative analyses of the lipid composition of human and great ape nervous and cardiovascular systems will be of special interest given the clinical phenotypes observed in humans with phytanic acid metabolic [43-45] and other peroxisomal disorders [86]. In addition, prostate tissue could be of interest given the differences of phytanic acid metabolic activities in normal and cancerous tissues [87-89] and male reproductive differences across human and non-human primates [90]. Lastly, we propose that larger-scale lipid profiling studies will likely identify additional candidate metabolic adaptations to human and non-human diets and that investigations into the microbial contributions toward specific metabolic differences is warranted.

## Methods

### Cohort for red blood cell lipid profiling

Bloods from adult humans with Western diets were collected from healthy individuals attending an international conference. Bloods from adult humans on vegan diets for over one year were collected in conjunction with a blood donor center. Appropriate Institutional Review Board (IRB) approval from the University of Southern California and Johns Hopkins Medicine was obtained for all human subjects research. Chimpanzee blood was collected at the Alamogordo Primate Facility. All chimpanzees took part in daily enrichment activities to maintain psychological well-being. Animals were maintained in accordance with the Guide for the Care and Use of Animals (U.S. Dept. of Health and Human Services, Public Health Service, Bethesda, MD., 1996). The APF and its program were fully accredited by the Association for Assessment and Accreditation of Laboratory Animal Care, International (AAALAC). Other great ape bloods were collected at the Zoological Society of San Diego. The gender and ages of blood donors are provided in Additional File 2. One gorilla blood donor, described in reference [91], carries a deletion of the distal q arm of chromosome 3 (the homolog of human chromosome 4) as indicated in Additional File 4. Great ape diets contain fresh fruits, vegetables, and nutritional biscuits. Capillary gas chromatography (GC) - electron-capture negative-ion mass spectrometry [92] analysis of nutritional biscuits indicated the great apes were exposed to <2 mg of daily phytanic acid intake.

### Red blood cell lipid profiling

Whole blood samples were collected from fasting individuals and stored in EDTA blood collection tubes. RBCs were collected by centrifugation, washed twice with physiological saline, transferred to freezer vials, flushed with nitrogen, and stored at -80°C until analysis. RBCs were thawed briefly before 100 μl aliquots were taken for analysis of the total lipid fatty acid content by capillary GC - electron-capture negative-ion mass spectrometry [92]. Processed data are provided in Additional File 4.

### Primary fibroblast cultures

Great ape dermal fibroblasts were obtained from the Zoological Society of San Diego while human dermal fibroblasts were obtained from the Coriell Institute for Medical Research or the Kennedy Krieger Institute. All individuals are thought to be unrelated. The gender, age, and biopsy site of all fibroblast donors and corresponding biochemical analyses are provided in Additional File 2. Fibroblasts were cultured as previously described [76].

### Phytanic and pristanic acid oxidation in cultured fibroblasts

Fatty acids were dried under nitrogen and solubilized with β-cyclodextrin (Sigma; 10 mg/ml in 10 mM Tris, pH 7.5) by warming to 37°C and sonication. Fibroblasts were grown to approximately 80% confluence in 12-well culture plates under standard conditions. Afterwards, the media was removed and replaced with 0.6 mL of serum-free culture media supplemented with 10 μM [1-^14^C]-phytanic acid (~40,000 dpm/nmol) or 10 μM [1-^14^C]-pristanic acid (~20,000 dpm/nmol). The cells were incubated for two hours (pristanic) or four hours (phytanic) at 37°C in 5% CO_2_. Reactions were terminated by addition of 0.12 ml of 2.6 N HClO_4_. Water-soluble reaction products were separated from the labeled substrates and quantified by liquid scintillation counting. The specific activity of phytanic acid or pristanic acid oxidation (pmoles/hour/mg protein) were calculated based on the amount of water-soluble radioactivity in the test sample minus the blank per mg of protein, determined using the Lowry method [93]. All primary data are provided in Additional File 5.

### Statistical considerations

We analyzed all data on the log2 scale. We used analysis of variance (ANOVA) to compare average blood lipid data across humans and great apes. Heterogeneity *P*-values are reported for the test that the mean level is different in at least one of the great apes groups, and Wald *P*-values for tests comparing the average level in individual non-human primate groups to humans. Under ANOVA, statistical tests use an estimate of within-group variation from all samples. Due to the unbalanced group sizes, this estimate is driven by the variation in humans and in chimpanzees. For gene expression studies, we tested for differential expression (Fig. 4) using ANOVA and standard F-tests. To account for testing differential expression across multiple genes, we adjusted p-values using the Benjamini and Hochberg approach for controlling the false-discovery rate (FDR). All analyses were done using the R programming language.

## Authors' contributions

PAW, ABM, CBT, and SJS carried out the biochemical analyses in this project. PAW, ABM, and HWM were involved in the design and conception of the peroxisome components of this project. MWK and KR maintained and conducted genetic and biochemical analysis on human and great ape cells. KDS conducted statistical analyses of all biochemical and gene expression data. RL and JJE provided characterized chimpanzee blood samples and diet information. OAR provided characterized great ape cells, blood samples, and diet information. OAR, JJE and RL were involved in the design and conception of the great ape components of the project. JGH was involved in the overall design and conception of the project, statistical analysis of all data sets, and wrote the manuscript with the help of all authors.

## Supplementary Material

Additional file 1 **Composition of blood donor cohort**. A summary of the numbers, ages, and sex of blood donors is provided. Click here for file

Additional file 2 **Skin fibroblast cultures used for phytanic and pristanic acid biochemical analysis**. A summary of donor sex and age and anatomical source of the skin fibroblasts is provided. Click here for file

Additional file 3 **Detailed summary of gene expression data for cross-species comparisons**. More complete gene expression data summary statistics relevant to Figure 4 are provided. Click here for file

Additional file 4 **Phytanic acid levels relative to total fatty acids in red blood cells from individual donors**. Relative phytanic acid levels for all RBC donors are provided. Click here for file

Additional file 5 **Rates of phytanic and pristanic acid oxidation in cultured dermal fibroblasts**. The rates of phytanic and pristanic acid oxidation from all individual fibroblast cultures are provided. Click here for file

## References

[B1] Mitchell P C. V. On the intestinal tract of mammals Trans Zool Soc Lond 1905 XVII 437 536

[B2] Milton K A hypothesis to explain the role of meat-eating in human evolution Evolutionary Anthropology 1999 8 1 11 21 10.1002/(SICI)1520-6505(1999)8:1<11::AID-EVAN6>3.0.CO;2-M

[B3] Milton K The critical role played by animal source foods in human (Homo) evolution J Nutr 2003 133 11 Suppl 2 3886S 3892S 14672286 10.1093/jn/133.11.3886S

[B4] Leonard WR Snodgrass JJ Robertson ML Effects of brain evolution on human nutrition and metabolism Annu Rev Nutr 2007 27 311 327 10.1146/annurev.nutr.27.061406.093659 17439362

[B5] Martin RD Chivers DJ Maclarnon AM Hladik CM Jungers WJ Gastrointestinal allometry in primates and other mammals Size and Scaling in Primate Biology 1985 Plenum: Plenum Press 61 139

[B6] Milton K Demment MW Digestion and passage kinetics of chimpanzees fed high and low fiber diets and comparison with human data J Nutr 1988 118 9 1082 1088 2843616 10.1093/jn/118.9.1082

[B7] Hladik CM Chivers DJ Pasquet P On Diet and Gut Size in Non-human Primates and Humans: Is There a Relationship to Brain Size? Curr Anthropol 1999 40 5 695 697 10.1086/300099 10539946

[B8] Leonard WR Robertson ML Aiello LC Wheeler P On diet, energy metabolism, and brain size in human evolution Current Anthropology 1996 37 125 129 10.1086/204476

[B9] Carmody RN Wrangham RW The energetic significance of cooking J Hum Evol 2009 57 4 379 391 10.1016/j.jhevol.2009.02.011 19732938

[B10] Schoeninger MJ Bunn HT Murray S Pickering T Moore J Stanford CB, Bunn HT Meat-eating by the fourth African ape Meat-eating and Human Evolution 2001 Oxford University Press. New York 179 195

[B11] Bunn HT Kroll EM Systematic butchery by Plio/Pleistocene hominids at Oldvai Gorge, Tanzania Current Anthropology 1986 27 431 452 10.1086/203467

[B12] Bunn HT Ungar PS Meat made us human Evolution of the human diet: The known, the unknown, and the unknowable 2006 Oxford: Oxford University Press 191 211

[B13] Marean CW Bar-Matthews M Bernatchez J Fisher E Goldberg P Herries AI Jacobs Z Jerardino A Karkanas P Minichillo T Early human use of marine resources and pigment in South Africa during the Middle Pleistocene Nature 2007 449 7164 905 908 10.1038/nature06204 17943129

[B14] Plummer T Flaked stones and old bones: biological and cultural evolution at the dawn of technology Am J Phys Anthropol 2004 Suppl 39 118 164 10.1002/ajpa.20157 15605391

[B15] Tishkoff SA Reed FA Ranciaro A Voight BF Babbitt CC Silverman JS Powell K Mortensen HM Hirbo JB Osman M Convergent adaptation of human lactase persistence in Africa and Europe Nat Genet 2007 39 1 31 40 10.1038/ng1946 17159977 PMC2672153

[B16] Rick TC Erlandson JM Anthropology. Coastal exploitation Science 2009 325 5943 952 953 10.1126/science.1178539 19696338

[B17] Richards MP Jacobi R Cook J Pettitt PB Stringer CB Isotope evidence for the intensive use of marine foods by Late Upper Palaeolithic humans J Hum Evol 2005 49 3 390 394 10.1016/j.jhevol.2005.05.002 15975629

[B18] Broadhurst CL Wang Y Crawford MA Cunnane SC Parkington JE Schmidt WF Brain-specific lipids from marine, lacustrine, or terrestrial food resources: potential impact on early African Homo sapiens Comp Biochem Physiol B Biochem Mol Biol 2002 131 4 653 673 10.1016/S1096-4959(02)00002-7 11923081

[B19] Shipman P Scavenging or hunting in early hominids: theoretical framework and tests American Anthropologist 1986 88 27 43 10.1525/aa.1986.88.1.02a00020

[B20] Rothman JM Dierenfeld ES Hintz HF Pell AN Nutritional quality of gorilla diets: consequences of age, sex, and season Oecologia 2008 155 1 111 122 10.1007/s00442-007-0901-1 17999090

[B21] Popovich DG Jenkins DJ Kendall CW Dierenfeld ES Carroll RW Tariq N Vidgen E The western lowland gorilla diet has implications for the health of humans and other hominoids J Nutr 1997 127 10 2000 2005 9311957 10.1093/jn/127.10.2000

[B22] Conklin-Brittain NL Knott CD Wrangham RW Hohmann G, Robbins MM, Boesch C Energy intake by wild chimpanzees and orangutans: methodological considerations and a preliminary comparison Feeding Ecology in Apes and Other Primates: Ecological, Physical, and Behavioral Aspects 2006 Cambridge: Cambridge University Press 445 472

[B23] Remis MJ Initial studies on the contributions of body size and gastrointestinal passage rates to dietary flexibility among gorillas Am J Phys Anthropol 2000 112 2 171 180 10.1002/(SICI)1096-8644(2000)112:2<171::AID-AJPA4>3.0.CO;2-F 10813700

[B24] Remis MJ Dierenfeld ES Digesta passage, digestibility, and behavior in captive gorillas under two dietary regimens International Journal of Primatology 2004 25 825 845 10.1023/B:IJOP.0000029124.04610.c7

[B25] Schmidt DA Kerley MS Dempsey JL Porton IJ Porter JH Griffin ME Ellersieck MR Sadler WC Fiber digestibility by the orangutan (Pongo abelii): in vitro and in vivo J Zoo Wildl Med 2005 36 4 571 580 10.1638/04-103.1 17312712

[B26] Cummings JH Englyst HN Fermentation in the human large intestine and the available substrates Am J Clin Nutr 1987 45 5 Suppl 1243 1255 3034048 10.1093/ajcn/45.5.1243

[B27] Mortensen PB Clausen MR Short-chain fatty acids in the human colon: relation to gastrointestinal health and disease Scand J Gastroenterol Suppl 1996 216 132 148 10.3109/00365529609094568 8726286

[B28] McNeil NI The contribution of the large intestine to energy supplies in man Am J Clin Nutr 1984 39 2 338 342 6320630 10.1093/ajcn/39.2.338

[B29] Gloerich J van Vlies N Jansen GA Denis S Ruiter JP van Werkhoven MA Duran M Vaz FM Wanders RJ Ferdinandusse S A phytol-enriched diet induces changes in fatty acid metabolism in mice both via PPARalpha-dependent and -independent pathways J Lipid Res 2005 46 4 716 726 10.1194/jlr.M400337-JLR200 15654129

[B30] Wolfrum C Ellinghaus P Fobker M Seedorf U Assmann G Borchers T Spener F Phytanic acid is ligand and transcriptional activator of murine liver fatty acid binding protein J Lipid Res 1999 40 4 708 714 10191295

[B31] Ellinghaus P Wolfrum C Assmann G Spener F Seedorf U Phytanic acid activates the peroxisome proliferator-activated receptor alpha (PPARalpha) in sterol carrier protein 2-/sterol carrier protein x-deficient mice J Biol Chem 1999 274 5 2766 2772 10.1074/jbc.274.5.2766 9915808

[B32] Goto T Takahashi N Kato S Egawa K Ebisu S Moriyama T Fushiki T Kawada T Phytol directly activates peroxisome proliferator-activated receptor alpha (PPARalpha) and regulates gene expression involved in lipid metabolism in PPARalpha-expressing HepG2 hepatocytes Biochem Biophys Res Commun 2005 337 2 440 445 10.1016/j.bbrc.2005.09.077 16202384

[B33] Heim M Johnson J Boess F Bendik I Weber P Hunziker W Fluhmann B Phytanic acid, a natural peroxisome proliferator-activated receptor (PPAR) agonist, regulates glucose metabolism in rat primary hepatocytes Faseb J 2002 16 7 718 720 11923221 10.1096/fj.01-0816fje

[B34] Lampen A Meyer S Nau H Phytanic acid and docosahexaenoic acid increase the metabolism of all-trans-retinoic acid and CYP26 gene expression in intestinal cells Biochim Biophys Acta 2001 1521 1-3 97 106 11690641 10.1016/s0167-4781(01)00305-0

[B35] Kitareewan S Burka LT Tomer KB Parker CE Deterding LJ Stevens RD Forman BM Mais DE Heyman RA McMorris T Phytol metabolites are circulating dietary factors that activate the nuclear receptor RXR Mol Biol Cell 1996 7 8 1153 1166 8856661 10.1091/mbc.7.8.1153 PMC275969

[B36] Schluter A Barbera MJ Iglesias R Giralt M Villarroya F Phytanic acid, a novel activator of uncoupling protein-1 gene transcription and brown adipocyte differentiation Biochem J 2002 362 Pt 1 61 69 10.1042/0264-6021:3620061 11829740 PMC1222360

[B37] Lemotte PK Keidel S Apfel CM Phytanic acid is a retinoid × receptor ligand Eur J Biochem 1996 236 1 328 333 10.1111/j.1432-1033.1996.00328.x 8617282

[B38] Radominska-Pandya A Chen G Photoaffinity labeling of human retinoid × receptor beta (RXRbeta) with 9-cis-retinoic acid: identification of phytanic acid, docosahexaenoic acid, and lithocholic acid as ligands for RXRbeta Biochemistry 2002 41 15 4883 4890 10.1021/bi0121151 11939783

[B39] van den Brink DM Wanders RJ Phytanic acid: production from phytol, its breakdown and role in human disease Cell Mol Life Sci 2006 63 15 1752 1765 10.1007/s00018-005-5463-y 16799769 PMC11136310

[B40] Wanders RJ Komen JC Peroxisomes, Refsum's disease and the alpha- and omega-oxidation of phytanic acid Biochem Soc Trans 2007 35 Pt 5 865 869 17956234 10.1042/BST0350865

[B41] Brown PJ Mei G Gibberd FB Burston D Mayne PD McClinchy JE Sidey M Diet and Refsum's disease. The determination of phytanic acid and phytol in certain foods and the application of this knowledge to the choice of suitable convenience foods for patients with Refsum's disease Journal of Human Nutrition and Dietetics 1993 6 295 305 10.1111/j.1365-277X.1993.tb00375.x

[B42] Avigan J The presence of phytanic acid in normal human and animal plasma Biochim Biophys Acta 1966 116 2 391 394 4163202 10.1016/0005-2760(66)90022-1

[B43] Steinberg D Stanbury JB, Wyngarden JB, Fredericksen DS, Goldstein JL, Brown MS Phytanic acid storage disease (Refsum's disease) Metabolic Basis of Inherited Disease 1983 5 New York: McGraw Hill 731 747

[B44] Wierzbicki AS Peroxisomal disorders affecting phytanic acid alpha-oxidation: a review Biochem Soc Trans 2007 35 Pt 5 881 886 17956237 10.1042/BST0350881

[B45] Verhoeven NM Wanders RJ Poll-The BT Saudubray JM Jakobs C The metabolism of phytanic acid and pristanic acid in man: a review J Inherit Metab Dis 1998 21 7 697 728 10.1023/A:1005476631419 9819701

[B46] Jansen GA Ofman R Ferdinandusse S Ijlst L Muijsers AO Skjeldal OH Stokke O Jakobs C Besley GT Wraith JE Refsum disease is caused by mutations in the phytanoyl-CoA hydroxylase gene Nat Genet 1997 17 2 190 193 10.1038/ng1097-190 9326940

[B47] Mihalik SJ Morrell JC Kim D Sacksteder KA Watkins PA Gould SJ Identification of PAHX, a Refsum disease gene Nat Genet 1997 17 2 185 189 10.1038/ng1097-185 9326939

[B48] Ferdinandusse S Zomer AW Komen JC van den Brink CE Thanos M Hamers FP Wanders RJ van der Saag PT Poll-The BT Brites P Ataxia with loss of Purkinje cells in a mouse model for Refsum disease Proc Natl Acad Sci USA 2008 105 46 17712 17717 10.1073/pnas.0806066105 19004801 PMC2584743

[B49] Moser AB Jones DS Raymond GV Moser HW Plasma and red blood cell fatty acids in peroxisomal disorders Neurochem Res 1999 24 2 187 197 10.1023/A:1022549618333 9972864

[B50] Coppack SW Evans R Gibberd FB Clemens ME Billimoria JD Can patients with Refsum's disease safely eat green vegetables? Br Med J (Clin Res Ed) 1988 296 6625 828 10.1136/bmj.296.6625.828 2453246 PMC2545156

[B51] Khaitovich P Hellmann I Enard W Nowick K Leinweber M Franz H Weiss G Lachmann M Paabo S Parallel patterns of evolution in the genomes and transcriptomes of humans and chimpanzees Science 2005 309 5742 1850 1854 10.1126/science.1108296 16141373

[B52] Toleno DM Renaud G Wolfsberg TG Islam M Wildman DE Siegmund KD Hacia JG Development and evaluation of new mask protocols for gene expression profiling in humans and chimpanzees BMC Bioinformatics 2009 10 1 77. 10.1186/1471-2105-10-77 19265541 PMC2660304

[B53] Atshaves BP McIntosh AL Landrock D Payne HR Mackie JT Maeda N Ball J Schroeder F Kier AB Effect of SCP-x gene ablation on branched-chain fatty acid metabolism Am J Physiol Gastrointest Liver Physiol 2007 292 3 G939 951 10.1152/ajpgi.00308.2006 17068117

[B54] Anderson SP Howroyd P Liu J Qian X Bahnemann R Swanson C Kwak MK Kensler TW Corton JC The transcriptional response to a peroxisome proliferator-activated receptor alpha agonist includes increased expression of proteome maintenance genes J Biol Chem 2004 279 50 52390 52398 10.1074/jbc.M409347200 15375163

[B55] Finch CE Stanford CB Meat-adaptive genes and the evolution of slower aging in humans Q Rev Biol 2004 79 1 3 50 10.1086/381662 15101252

[B56] Goodall J The chimpanzees of Gombe: patterns of behavior 1986 Cambridge, Mass.: Belknap Press of Harvard University Press

[B57] Stanford CB Chimpanzee and red colobus: the ecology of predator and prey 1998 Cambridge, Mass.: Harvard University Press

[B58] Stanford CB Wallis J Matama H Goodall J Patterns of predation by chimpanzees on red colobus monkeys in Gombe National Park, 1982-1991 Am J Phys Anthropol 1994 94 2 213 228 10.1002/ajpa.1330940206 8085613

[B59] Boesch C Boesch H Hunting behavior of wild chimpanzees in the Tai National Park Am J Phys Anthropol 1989 78 4 547 573 10.1002/ajpa.1330780410 2540662

[B60] Steinberg D Mize CE Herndon JH Fales HM Engel WK Vroom FQ Phytanic acid in patients with Refsum's syndrome and response to dietary treatment Arch Intern Med 1970 125 1 75 87 10.1001/archinte.125.1.75 4188898

[B61] Gootjes J Mooijer PA Dekker C Barth PG Poll-The BT Waterham HR Wanders RJ Biochemical markers predicting survival in peroxisome biogenesis disorders Neurology 2002 59 11 1746 1749 12473763 10.1212/01.wnl.0000036609.14203.70

[B62] Steinberg D Avigan J Mize CE Baxter JH Cammermeyer J Fales HM Highet PF Effects of dietary phytol and phytanic acid in animals J Lipid Res 1966 7 5 684 691 4165840

[B63] Allen NE Grace PB Ginn A Travis RC Roddam AW Appleby PN Key T Phytanic acid: measurement of plasma concentrations by gas-liquid chromatography-mass spectrometry analysis and associations with diet and other plasma fatty acids Br J Nutr 2008 99 3 653 659 10.1017/S000711450782407X 17868488

[B64] Aiello LC Notes on the implications of the expensive tissue hypothesis for human biological and social evolution Guts and brains 2007 Roebroeks W: Amsterdam University Press 17 28

[B65] Aiello LC Wheeler P The expensive-tissue hypothesis: The brain and the digestive system in human and primate evolution Current Anthropology 1995 36 2 199 221 10.1086/204350

[B66] Ley RE Lozupone CA Hamady M Knight R Gordon JI Worlds within worlds: evolution of the vertebrate gut microbiota Nat Rev Microbiol 2008 6 10 776 788 10.1038/nrmicro1978 18794915 PMC2664199

[B67] Blaser MJ Falkow S What are the consequences of the disappearing human microbiota? Nat Rev Microbiol 2009 7 12 887 894 10.1038/nrmicro2245 19898491 PMC9354563

[B68] Mittendorfer B Sexual dimorphism in human lipid metabolism J Nutr 2005 135 4 681 686 15795418 10.1093/jn/135.4.681

[B69] Kitson AP Stroud CK Stark KD Elevated production of docosahexaenoic acid in females: potential molecular mechanisms Lipids 2010 45 3 209 224 10.1007/s11745-010-3391-6 20151220

[B70] Atshaves BP Payne HR McIntosh AL Tichy SE Russell D Kier AB Schroeder F Sexually dimorphic metabolism of branched-chain lipids in C57BL/6J mice J Lipid Res 2004 45 5 812 830 10.1194/jlr.M300408-JLR200 14993239

[B71] Roff CF Pastuszyn A Strauss JF Billheimer JT Vanier MT Brady RO Scallen TJ Pentchev PG Deficiencies in sex-regulated expression and levels of two hepatic sterol carrier proteins in a murine model of Niemann-Pick type C disease J Biol Chem 1992 267 22 15902 15908 1639819

[B72] McLean MP Billheimer JT Warden KJ Irby RB Differential expression of hepatic sterol carrier proteins in the streptozotocin-treated diabetic rat Endocrinology 1995 136 8 3360 3368 10.1210/en.136.8.3360 7628371

[B73] Blekhman R Marioni JC Zumbo P Stephens M Gilad Y Sex-specific and lineage-specific alternative splicing in primates Genome Res 2010 20 2 180 189 10.1101/gr.099226.109 20009012 PMC2813474

[B74] Stokke O Alpha-oxidation of fatty acids in various mammals, and a phytanic acid feeding experiment in an animal with a low alpha-oxidation capacity Scand J Clin Lab Invest 1967 20 305 312 10.3109/00365516709076960

[B75] Blekhman R Oshlack A Chabot AE Smyth GK Gilad Y Gene regulation in primates evolves under tissue-specific selection pressures PLoS Genet 2008 4 11 e1000271 10.1371/journal.pgen.1000271 19023414 PMC2581600

[B76] Karaman MW Houck ML Chemnick LG Nagpal S Chawannakul D Sudano D Pike BL Ho VV Ryder OA Hacia JG Comparative analysis of gene-expression patterns in human and african great ape cultured fibroblasts Genome Res 2003 13 7 1619 1630 10.1101/gr.1289803 12840040 PMC403735

[B77] Lammey ML Lee DR Ely JJ Sleeper MM Sudden cardiac death in 13 captive chimpanzees (Pan troglodytes) J Med Primatol 2008 37 Suppl 1 39 43 10.1111/j.1600-0684.2007.00260.x 18269527

[B78] Haygood R Fedrigo O Hanson B Yokoyama KD Wray GA Promoter regions of many neural- and nutrition-related genes have experienced positive selection during human evolution Nat Genet 2007 39 9 1140 1144 10.1038/ng2104 17694055

[B79] Somel M Creely H Franz H Mueller U Lachmann M Khaitovich P Paabo S Human and chimpanzee gene expression differences replicated in mice fed different diets PLoS ONE 2008 3 1 e1504. 10.1371/journal.pone.0001504 18231591 PMC2200793

[B80] O'Connell MJ McInerney JO Adaptive evolution of the human fatty acid synthase gene: support for the cancer selection and fat utilization hypotheses? Gene 2005 360 2 151 159 10.1016/j.gene.2005.06.020 16154299

[B81] Horrobin DF Lipid metabolism, human evolution and schizophrenia Prostaglandins Leukot Essent Fatty Acids 1999 60 5-6 431 437 10.1016/S0952-3278(99)80024-6 10471133

[B82] O'Connell JF Hawkes K Blurton Jones NG Grandmothering and the evolution of homo erectus J Hum Evol 1999 36 5 461 485 10.1006/jhev.1998.0285 10222165

[B83] Erren TC Erren M Can fat explain the human brain's big bang evolution?-Horrobin's leads for comparative and functional genomics Prostaglandins Leukot Essent Fatty Acids 2004 70 4 345 347 10.1016/j.plefa.2003.12.008 15041025

[B84] Varki A Multiple changes in sialic acid biology during human evolution Glycoconj J 2009 26 3 231 245 10.1007/s10719-008-9183-z 18777136 PMC7087641

[B85] Finch CE Evolution in health and medicine Sackler colloquium: Evolution of the human lifespan and diseases of aging: roles of infection, inflammation, and nutrition Proc Natl Acad Sci USA 2010 107 Suppl 1 1718 1724 10.1073/pnas.0909606106 19966301 PMC2868286

[B86] Yik WY Steinberg SJ Moser AB Moser HW Hacia JG Identification of novel mutations and sequence variation in the Zellweger syndrome spectrum of peroxisome biogenesis disorders Hum Mutat 2009 30 3 E467 480 10.1002/humu.20932 19105186 PMC2649967

[B87] Xu J Thornburg T Turner AR Vitolins M Case D Shadle J Hinson L Sun J Liu W Chang B Serum levels of phytanic acid are associated with prostate cancer risk Prostate 2005 63 3 209 214 10.1002/pros.20233 15712232

[B88] Zha S Ferdinandusse S Hicks JL Denis S Dunn TA Wanders RJ Luo J De Marzo AM Isaacs WB Peroxisomal branched chain fatty acid beta-oxidation pathway is upregulated in prostate cancer Prostate 2005 63 4 316 323 10.1002/pros.20177 15599942

[B89] Magda D Lecane P Prescott J Thiemann P Ma X Dranchak PK Toleno DM Ramaswamy K Siegmund KD Hacia JG mtDNA depletion confers specific gene expression profiles in human cells grown in culture and in xenograft BMC Genomics 2008 9 521 10.1186/1471-2164-9-521 18980691 PMC2612029

[B90] Dixson AF Primate sexuality: comparative studies of the prosimians, monkeys, apes, and human beings 1998 Oxford; New York: Oxford University Press

[B91] Lear TL Houck ML Zhang YW Debnar LA Sutherland-Smith MR Young L Jones KL Benirschke K Trisomy 17 in a bonobo (Pan paniscus) and deletion of 3q in a lowland gorilla (Gorilla gorilla gorilla): comparison with human trisomy 18 and human deletion 4q syndrome Cytogenet Cell Genet 2001 95 3-4 228 233 10.1159/000059350 12063404

[B92] Lagerstedt SA Hinrichs DR Batt SM Magera MJ Rinaldo P McConnell JP Quantitative determination of plasma c8-c26 total fatty acids for the biochemical diagnosis of nutritional and metabolic disorders Mol Genet Metab 2001 73 1 38 45 10.1006/mgme.2001.3170 11350181

[B93] Lowry OH Rosebrough NJ Farr AL Randall RJ Protein measurement with the Folin phenol reagent J Biol Chem 1951 193 1 265 275 14907713

